# Synthesis of a Smart Conductive Block Copolymer Responsive to Heat and Near Infrared Light

**DOI:** 10.3390/polym11111744

**Published:** 2019-10-24

**Authors:** Silvestre Bongiovanni Abel, Kevin Riberi, Claudia R. Rivarola, Maria Molina, Cesar A. Barbero

**Affiliations:** 1 Research Institute for Energy Technologies and Advanced Materials (IITEMA), National University of Río Cuarto (UNRC)-National Council of Scientific and Technical Research (CONICET), Ruta Nacional N° 36, Km 601, Río Cuarto (Córdoba) 5800, Argentina; bongiovanniabel.s@fi.mdp.edu.ar (S.B.A.); kriberi@exa.unrc.edu.ar (K.R.); crivarola@exa.unrc.edu.ar (C.R.R.); 2 Research Institute of Materials Science and Technology (INTEMA), National University of Mar del Plata (UNMdP)-National Council of Scientific and Technical Research (CONICET), Av. Colón 10850, B7608FDQ, Mar del Plata (Buenos Aires) 7600, Argentina

**Keywords:** block copolymer, conductive polymer, thermal response, near infrared light, photothermal behavior, poly(*N*-isopropylacrylamide), polyaniline

## Abstract

A method for the synthesis of a linear block copolymer (PNIPAM-*b*-PANI), containing a thermoresponsive block (poly(*N*-isopropylacrylamide), PNIPAM) and a Near Infrared (NIR) light-absorbing block (polyaniline, PANI), is reported. The synthetic approach involves a two-step successive polymerization reaction. First, the radical polymerization of NIPAM is done using 4-aminothiophenol as a chain transfer agent for the obtention of thermosensitive block terminated with an aniline (ANI) moiety. Second, the oxidative polymerization of ANI is initiated in ANI moiety of thermosensitive block to grow the second conductive PANI block. ^1^H nuclear magnetic resonance (NMR) and FT-IR spectroscopy shows the characteristics peaks of both polymeric blocks revealing the successful copolymerization process. Static Light Scattering (SLS) and UV-Visible combined measurements allowed the determination of the *M*_w_ for PNIPAM-*b*-PANI macromolecule: 5.5 × 10^5^ g mol^−1^. The resulting copolymer is soluble in water (8.3 g L^−1^) and in non-aqueous solvents, such as ethanol, formic acid, acetonitrile, and others. Both polymer blocks chains show the properties of the polymer chains. The block copolymer shows a lower critical solution temperature (LCST) at the same temperature (32–34 °C) than PNIPAM, while the copolymer shows pH dependent UV-vis-NIR absorption similar to PANI. The PNIPAM block suffers a coil to globule transition upon NIR light irradiation (785 nm, 100 mW), as shown by turbidimetry and Atomic Force Microscopy (AFM), due to local heating (more than 9 °C in 12 min) induced by the NIR absorption at the PANI block. Furthermore, the electrical conductivity of PNIPAM-*b*-PANI thin films is demonstrated (resistivity of 5.3 × 10^−4^ Ω^−1^ cm^−1^), indicating that the PANI block is present in its conductive form.

## 1. Introduction

Conductive polymers (CPs) have attracted the attention of the scientific and technology community due to their electrochemical, electronic, and optical properties [1,2]. CPs have been also applied in nanomedicine [3], specifically as photothermal absorbers for anticancer and antibacterial photothermal therapy [4,5,6]. This is possible because CPs absorb electromagnetic radiation and transform it in heat, inducing tumor cell or bacteria death [7,8].

Polyaniline (PANI) is the most commonly used CP because of its thermal stability, low cost, and versatility [9]. However, the insolubility in common solvents and biological fluids is its main disadvantage [10]. Therefore, PANI could not reach the tumor cells to provoke the photothermal killing. Different strategies have been used to disperse or solubilize this CP. Some examples include chemical functionalization [11] or preparation of nanoparticles as stable dispersions in water [12,13]. We propose a new alternative, namely the block copolymerization of PANI with a water-soluble polymer chain.

Poly(*N*-isopropylacrylamide) (PNIPAM) is a well-studied water soluble and thermoresponsive polymer which presents a lower critical solution temperature (LCST) at ca. 32–34 °C [14]. 

In a previous work [15], PANI nanoparticles were stabilized with a water-soluble thermosensitive copolymer containing *N*-isopropylacrylamide (NIPAM) monomer units. The smart nanoparticles aggregated upon heating or NIR light irradiation by the local photothermal effect. Other kinds of applications were the combination of those polymers to generate macroscopic composites to be used as pressure sensors and thermal switch, microwave responsive devices, and chemo-mechanical actuators [16,17,18]. In recent years, a series of copolymers based on PNIPAM were synthesized, demonstrating the versatility of this thermosensitive polymer for the development of new materials [19,20,21,22,23]. Hsiao et al. [24] demonstrated that it is possible to grow PANI from the alkyl amine (R-NH_2_) groups of chitosan producing another kind of NIR absorbing hydrogel. 

In this work, a block copolymer (PNIPAM-*b*-PANI) made of a thermosensitive (PNIPAM) and a conductive (PANI) block is synthesized by a novel approach. First, PNIPAM polymer terminated with aniline moieties is generated by radical vinyl polymerization using an appropriate charge transfer agent (4-aminothiophenol, 4-ATF). In a second step, the PANI block is grown by oxidative polymerization of aniline (ANI), which initiates in the extreme of the PNIPAM block that contains the aniline group, in order to link the PNIPAM chains with PANI. The copolymer is soluble in aqueous solutions and some common solvents due to the effect of the soluble PNIPAM block. The obtained copolymer is characterized by FT-IR and NMR. The molecular weight determined by a combination of Static Light Scattering (SLS) and UV-visible spectroscopy.

The copolymer clearly aggregates in water when the temperature of the solution goes above 34 °C, showing that the thermosensitive behavior of the PNIPAM block is preserved. At the same time, the copolymer shows the typical UV-visible spectra of PANI, which presents a broad band at ca. 830 nm. Finally, when the solution is illuminated with NIR light (780 nm), the copolymer also aggregates. This behavior is possible because the PANI block absorbs the NIR light, locally heats the environment of the copolymer, and the phase transition of the PNIPAM block takes place when the LCST is overcome. In that way, the copolymer constitutes the smallest phase transition device induced photothermally. 

## 2. Materials and Methods 

### 2.1. Materials

*N*-isopropylacrylamide (NIPAM) used as monomer was obtained from Scientific Polymer Products (Wayne County, NY, USA). 2,2′-azobis(2-(2-imidazolin-2-yl)propane] dihydrochloride (VA044), ammonium peroxydisulfate (APS), and 4-aminothiophenol (4-ATF) were purchased from Sigma-Aldrich (St. Louis, MO, USA). HCl and methanol were obtained from Anedra. Anilinium hydrochloride (ANI) was purchased from Merck (Kenilworth, NJ, USA). Water used in this work was triply distilled. All reagents were analytical quality and used as received.

### 2.2. Synthesis of PNIPAM-b-PANI

The first synthetic step was carried out using a 1 mol L^−1^ NIPAM solution in water and VA044 (5 × 10^−3^ g mL^−1^) as thermally activated initiator agent of polymerization. Temperature (60 ± 0.1 °C), stirring conditions, and gas bubbling (N_2_) were kept during 4 h to complete the polymerization process. 4-ATF was used as chain transfer agent at 3 × 10^−5^ mol L^−1^ [25]. The aniline terminated PNIPAM molecule was purified by several heating–cooling cycles and centrifugation to remove remaining reactants or unreacted monomer. Then, the polymer was precipitated in methanol, obtaining the ATF terminated PNIPAM (PNIPAM-ATF) as a white solid.

Using the modified thermoresponsive block in water, the polymerization of ANI was carried out. 15 mL of 0.1 mol L^−1^ of ANI in 1 mol L^−1^ HCl (prepared and maintained at 0 ± 0.1 °C) was added to PNIPAM-ATF and stirred to obtain a clear solution. Then, APS (equimolar to ANI) was added as an oxidant agent to initiate the polymerization. The conducting polymer block grows from the terminal ANI groups at the modified PNIPAM molecules. In this way, a green solution of PNIPAM-*b*-PANI was obtained. Because PNIPAM is a vinylic chain polymer, no chain scission is expected by the effect of persulfate ions. Indeed, aniline polymerization inside PNIPAM gels is a well-established method, and no evidence of chain scission has been observed [26].

Finally, several heating–cooling cycles were carried out to purify the synthesized copolymer. In each cycle, the copolymer is aggregated by heating at 40 °C and separated by centrifugation. The supernatant discarded contain soluble reactants and side products, together with PNIPAM blocks not attached to PANI which suffers the coil to globule transition above the LCST but remains soluble, unlike the copolymer which contains the water insoluble PANI group. In that way, only block copolymer molecules are present in the solid. The purified precipitate is dried by freeze drying. 

### 2.3. Solubility

Solubility of PNIPAM-*b*-PANI was tested at 20 °C in different solvents: 1-octanol (S. Aldrich, for High Performance Liquid Chromatography (HPLC) ≥ 99%), acetone (Anedra, 99.5%), acetonitrile (S. Aldrich, ACS Reagent ≥ 99.5%), carbon tetrachloride (S. Aldrich, anhydrous ≥ 99.5%), chloroform (Sintorgan, Buenos Aires, Argentina, HPLC grade), cyclohexane (Sintorgan, HPLC grade), cyclohexanol (S. Aldrich, ACS Reagent ≥ 99.5%), dichloromethane (S. Aldrich, anhydrous ≥ 99.8%), dimethyl sulfoxide (S. Aldrich, ACS Reagent ≥ 99.5%), ethanol (Sintorgan, HPLC grade), formic acid (Cicarelli, Pro-Analysis), isopropyl alcohol (S. Aldrich, ≥ 99.7%), *N*-methyl-2-pyrrolidone (S. Aldrich, ≥ 99.5%), pyridine (S. Aldrich, anhydrous ≥ 99.8%), tetrahydrofuran (S. Aldrich, anhydrous ≥ 99.9%), and water (Milli-Q quality, triply distilled). The solubility test was made by weighing a known mass (ca. 1 × 10^−2^ g) of the lyophilized copolymer and added to 2 mL of the chosen solvents. A Vortex device was used to help the solubilization process. By direct observation of the dissolution after 24 h, the samples were classified considering turbidity and precipitates as: non-soluble (no color in solution, colored precipitate presence), slightly soluble (cloudy solutions or particle presence), and soluble (clear colored solution, no precipitate presence).

### 2.4. Characterization Techniques

#### 2.4.1. Molecular Weight (*M*_w_) Determination

The *M*_w_ of the copolymer could not be determined by Gel Permeation Chromatography (GPC) and Static Light Scattering (SLS) due to the aggregation of PANI chains in usual GPC solvents. Therefore, a procedure was developed to determine the molecular weight (*M*_w_) in two steps. First, the *M*_w_ of the PNIPAM block (terminated by aniline group) was determined by SLS measurements. A commercial SLS Malvern 4700 spectrometer (Malvern Instruments, Malvern, UK) equipped with a 488 nm laser OBIS was used. The details of instrumentation and theories have been described elsewhere. All measurements were carried out at 25 °C. The specific refractive index increments (dn/dc) of PNIPAM in water at 25 °C were determined using a differential refractometer (BI-DCP, Brookhaven Instruments, New York, NY, USA). The measured dn/dc values of thermosensitive block at 25 °C and 480 nm in water was 0.1332 mL g^−1^. The mass of the conductive block in the copolymer was determined by UV-Visible spectroscopy measurements using a HP 8453 (Hewlett Packard, Palo Alto, CA, USA). The mass absorption coefficient of pure PANI (emeraldine base state) in *N*-methyl-2-pyrrolidone (NMP) was determined measuring the optical absorption at 328 nm. It is assumed that the absorption coefficient of the PANI block is the same. 

The *M*_w_ was calculated using the following equations:

(1) $$m_{PNIPAM}^{copol}=m_{copol}-m_{PANI}^{copol},$$

where $m_{PNIPAM}^{copol}$ is the mass of PNIPAM block, $m_{copol}$ is the mass of copolymer, and $m_{PANI}^{copol}$ is the mass of PANI block. 

The number of PNIPAM chains was determined using the *M*_w_ of the PNIPAM block and assuming 100% efficiency of the initiation by the aniline moieties of the PANI block formation; all PNIPAM chains have PANI chains attached, and the number of copolymer chains was calculated by Equation (2). It is not likely that 100% of the PNIPAM blocks become attached to PANI blocks, but the synthetic procedure eliminates free PANI chains, which are insoluble in water, and also free PNIPAM block chains, which remain in solution when the copolymer is purified by aggregation promoted by heating since PNIPAM suffers transition but remains soluble

(2) $$N_{chains}^{copol}=N_{chains}^{PNIPAM}=\frac{m_{PNIPAM}}{Mw_{PNIPAM}}.$$

In this way, the *M*_w_ of the copolymer and the *M*_w_ of the PANI block was calculated.

(3) $$Mw_{copol}=\frac{m_{copol}}{N_{chains}^{copol}},$$

(4) $$Mw_{PANI}=Mw_{copol}-Mw_{PNIPAM}.$$

#### 2.4.2. Proton Nuclear Magnetic Resonance (^1^H NMR)

The synthesized macromolecule was fully characterized by Proton Nuclear Magnetic Resonance (^1^H NMR) in deuterated water. Samples were prepared drying the polymer in a vacuum device at 50 ± 0.1 °C during 48 h. Then, the copolymer was re-dissolved using deuterated water to analyze in the spectrometer. Proton nuclear magnetic resonance spectra were performed using a FT-NMR Bruker Ultra Shield 400 spectrometer (Bruker, Billerica, MA, USA) at 400 MHz 1H frequency. 

#### 2.4.3. Fourier Transformed Infrared Spectroscopy (FT-IR)

Fourier Transform Infrared Spectroscopy measurements were recorded in ATR mode on a FT-IR Perkin Elmer Model Spectrum Two (Perkin Elmer, Waltham, MA, USA. The device was equipped with a DynaScan interpherometer. Software used as the interface was Spectrum 10. The spectrum was recorder from 600 to 2000 cm^−1^ with a resolution of 4 cm^−1^, setting 64 scans. Air was used as background, and the measurement was made at room temperature. The block copolymer sample (in conductive form, doped) was previously lyophilized and dried under vacuum at room temperature for 48 h. 

#### 2.4.4. UV-Visible Spectroscopy and Turbidimetry Measurements

A Hewlett-Packard 8453 diode array spectrophotometer was used to obtain UV-Visible spectra. Samples were diluted using triply distilled water. The spectra were taken in quartz cells (0.1 cm path length). The spectra of the doped and undoped copolymer were measured using buffers to control pH of the sample (doped: pH 4; undoped: pH 10). The wavelength range for measurement was 300–900 nm. Turbidimetry was determined by measuring the absorption at a wavelength of 600 nm in quartz cells. Temperature was set up in the range between 25–42 °C by heating in water baths. 

#### 2.4.5. Conductivity Test

The resistance measurement was made on an interdigital copper electrode with small gap (width = 400 µm) between fingers. A PNIPAM-*b*-PANI drop was deposited on the electrode. The resistance was quantified as time function using a DC multimeter and a Bs20x Data Recording System V4.x as the interface program. The thickness of the film was estimated from the mass of copolymer deposited. The area of the deposit was measured from the calibrated photograph, and the density of the copolymer was determined with a pycnometer. 

#### 2.4.6. Photothermal Effect

In testing the photothermally induced effect, free standing solution drops (5 × 10^−3^ g L^−1^) of PNIPAM-*b*-PANI in water were irradiated by a NIR laser (780 nm and 100 mW power) through a fiber optic. Temperature of the drops was monitored by a TES 1326S/1327K infrared thermometer calibrated with a type K thermocouple. Duration of experiment was 15 min, recording the temperature at one-minute intervals, as previously described [15]. A thermographic camera Ti10 IR-Fusion Technology (Fluke, Everett, WA, USA) was used to capture IR images. Digitalization was made with a SmartView^®^ software. 

#### 2.4.7. Atomic Force Microscopy (AFM)

AFM has been used successfully to study the morphology of block copolymers film [27,28,29,30]. Here, we used to monitor the reversible changes of microstructure induced by the triggering of a coil to globule transition of PNIPAM blocks. An atomic force Agilent 5420 AFM/STM microscope (Agilent, Santa Clara, CA, USA) was used to take measurements of thin films before and after irradiate the macromolecule using the NIR laser. Drops (ca. 5 × 10^−3^ g L^−1^) were deposited on flat mica surfaces, and dried in air before scanning. A Point Probe^®^ Plus Non-Contact/Tapping Mode-Long Cantilever (PPP NCL) with a force constant of 6 N m^−1^ and resonance frequency of 156 Hz was used in the Acoustic AC (AAC) mode. Images were taken before and after two min of NIR irradiation.

## 3. Results and Discussion

**Figure 1:**
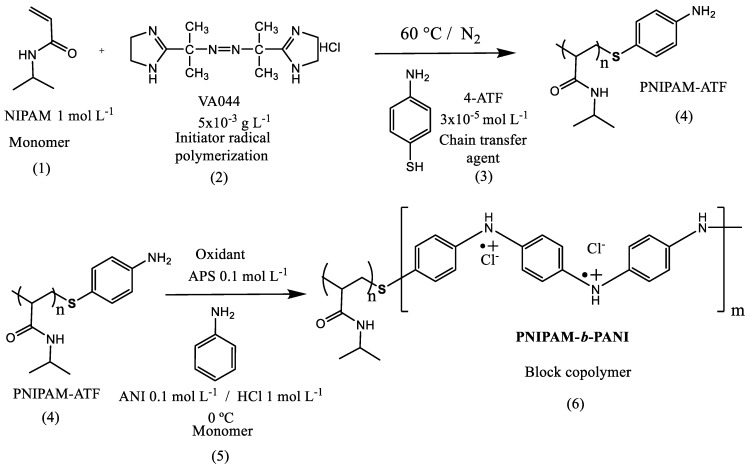
Schematic representation of the PNIPAM-*b*-PANI synthesis. PNIPAM = Poly(*N*-isopropylacrylamide); PANI = polyaniline; ATF = aminothiophenol; ANI = aniline; APS = ammonium peroxydisulfate.

The block copolymer based on PNIPAM and PANI was synthesized in two steps (Figure 1). First, a PNIPAM chain terminated in aniline unit was obtained by free radical polymerization. In this sense, the NIPAM (1) monomer is used to polymerize using an azo initiator (2) in the presence of a chain transfer agent (4-ATF) (3) to yield PNIPAM chains terminated by aniline groups (4, PNIPAM-ATF) [25]. This reaction is followed by the oxidative polymerization of aniline (5) monomer with APS as oxidant. The PANI chain grows from the –S–C_6_H_4_–NH_2_ extreme of PNIPAM block. This is a well-known method to grow PANI chains attached to other polymer chains [31] or surfaces [32]. In that way, the linear block copolymer (PNIPAM-*b*-PANI) (6) is obtained.

**Figure 2:**
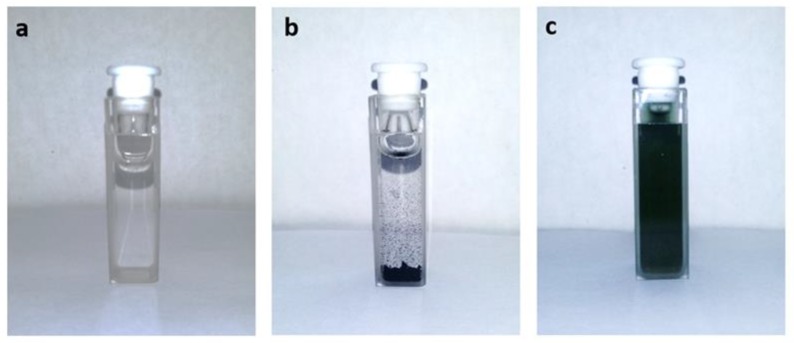
Photographs of optical cells containing aqueous solutions of: (**a**) PNIPAM, (**b**) PANI, and (**c**) PNIPAM-*b*-PANI at 20 °C.

PNIPAM chains are soluble in water and do not absorb in the UV-visible range, while PANI chains are insoluble and strongly colored (green in acid media). The copolymer obtained as a green water-soluble powder. The solubility in water results 8.3 g L^−1^. Photographs of each block (PNIPAM and PANI) and the resulting material (PNIPAM-*b*-PANI) are shown in Figure 2. 

**Figure 3:**
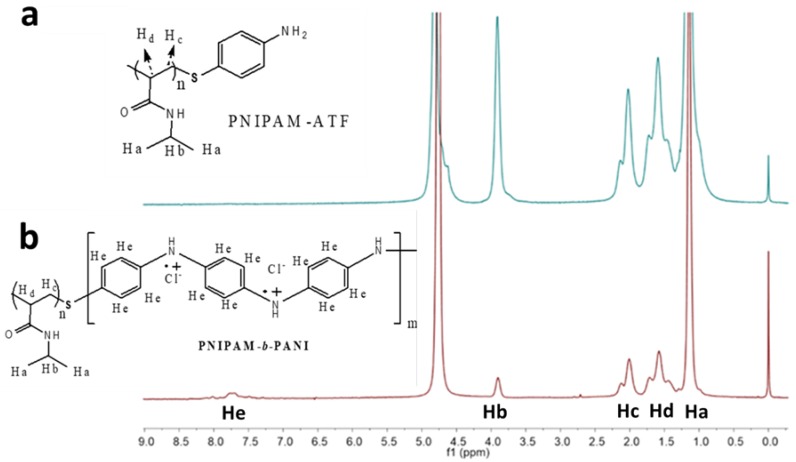
^1^H nuclear magnetic resonance (NMR) spectra: (**a**) PNIPAM-ATF and (**b**) PNIPAM-*b*-PANI.

The copolymer structure was studied by ^1^H NMR (Figure 3). ^1^H NMR of PNIPAM-ATF shows following peaks: δ: 1.13 (s, 6H, isopropyl groups of NIPAM), 1.57 (2H, polymer backbone), 2.00 (1H, polymer backbone), and 3.70–4.00 (1H, NIPAM). The results are in agreement with previous study of PNIPAM using NMR [33]. All the aforementioned peaks and a new signal at ~7.7 ppm are observed [13]. ^1^H NMR PNIPAM-*b*-PANI: δ: 1.13 (s, 6H, isopropyl groups of NIPAM), 1.57 (2H, polymer backbone), 2.00 (1H, polymer backbone), 3.70–4.00 (1H NIPAM), and 7.50–8.00 (4H, aromatic protons of PANI).

The broad signal due to aromatic protons in PANI is quite weak. It is likely that the rigid nature of the PANI chains broadens the signal and decreases the intensity [34]. Therefore, it cannot be used to calculate the copolymer composition but reveals that aniline monomer units are present in the copolymer. 

**Figure 4:**
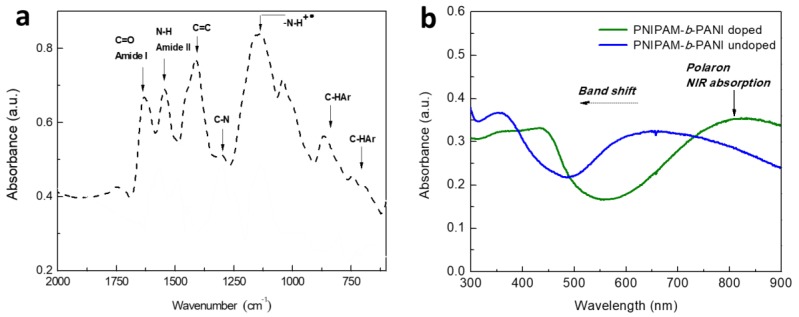
(**a**) FTIR spectrum for doped copolymer; (**b**) UV-Visible spectra in acidic and basic media of PNIPAM-*b*-PANI.

The FT-IR spectrum for PNIPAM-*b*-PANI is shown in Figure 4a. After a thorough analysis of the main bands, the assignation is following detailed: (i) the presence of band at ca. 1410 cm^−1^ corresponds to C=C; (ii) the band corresponding to the C–N stretching is present at 1298 cm^−1^; (iii) the bands between 750–900 cm^−1^ are referred to C–H of Ar present in the PANI backbone; (iv) the prominent band at 1140 cm^−1^ is in agreement with the reported by several authors for the vibrational mode of protonated amines (–N–H^+^) generated during the acid doping process of the CP [35,36]; (v) the presence of the N–H bending corresponding to the amide II of PNIPAM is detected at 1530 cm^−1^; and (vi) the C=O stretching of amide I of the thermosensitive block appears at 1625 cm^−1^ [37]. 

The UV-Visible spectra show the electronic transitions of the PANI block (Figure 4b) which change upon exposure to acid or basic media in agreement with previous results for PANI nanostructures, such as films, nanofibers, and nanospheres [38,39]. As expected, the pH sensitivity of the PANI block is unaffected by the presence of the PNIPAM block. This result is relevant for photothermal therapy applications since the spectra show absorption in the NIR range (>750 nm) [40,41,42]. Besides, it allows the building of sensors/actuators devices that respond to UV-Visible and NIR spectral range.

The *M*_w_ was determined in two steps, as described before. First, the *M*_w_ of the PNIPAM block was determined by SLS measurements, giving a value of 3.8 × 10^5^ g mol^−1^ (degree of polymerization 3360). In a second step, the *M*_w_ of the conductive block was calculated to be 1.7 × 10^5^ g mol^−1^ (degree of polymerization = 1870). This value is in good agreement with the reported ones for the molecular weight of chemically synthesized PANI measured by GPC by Wei and coworkers [43]. Adding these values, we could estimate that the block copolymer has a *M*_w_ of 5.5 × 10^5^ g mol^−1^. 

The block copolymer turned out to be soluble in several aqueous and organic solvents where PNIPAM is soluble but PANI is insoluble (Table 1). Obviously, the copolymer is soluble in those solvents marked with # in Table 1 where both PNIPAM and PANI are soluble. Since only solubility in water, where PNIPAM shows a clear coil to globule transition, is required to achieve the goal of the paper, no attempt was made to increase the solubility.

**Figure 5:**
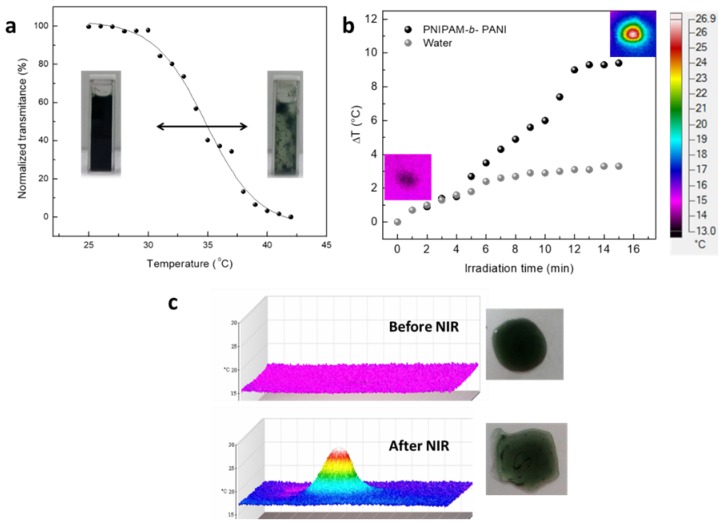
Material characterization of PNIPAM-*b*-PANI. (**a**) Turbidimetry as temperature function in aqueous solution; (**b**) photothermal effect on drop of PNIPAM-*b*-PANI aqueous solution under Near Infrared (NIR) irradiation; (**c**) optical and 3D infrared photograph of an aqueous drop containing the copolymer before and after NIR irradiation.

The turbidity of a PNIPAM-*b*-PANI aqueous solution is studied as a function of the increasing temperature (Figure 5a). The transmittance decreases upon an increase of temperature with an inflection point at ~34 °C. This value is close to the LCST of PNIPAM [19]. At lower temperatures, the PNIPAM block is extended in the solution interacting by hydrogen bonding with water, making the copolymer soluble. At temperatures higher than LCST, the PNIPAM block becomes globular decreasing its interaction with water and making the copolymer insoluble. Therefore, the solution became cloudy, decreasing the transmittance due to increased dispersion. This change upon heating confirms the presence of a PNIPAM block covalently linked to the PANI block. It is important to remark the total reversibility of the behavior when the room temperature is newly achieved. This fact could allow the fabrication of a thermal switch at the molecular level. The photograph inserted in Figure 5a confirms the observed changes for thermally induced polymer aggregation.

The photothermal effect by NIR irradiation was also studied. The temperature of a copolymer aqueous solution at pH 4 was measured during NIR irradiation (785 nm laser of 100 mW power). For this purpose, a NIR laser was directed onto a free-standing solution drops on glass using an optical fiber. The results are shown in Figure 5b,c. It can be seen that the temperature of drop increases with time when NIR irradiation is applied, reaching an increase of ~9 °C after 12 min. The same experience applied to pure water drop shows an increment of only 3 °C under the same irradiation time. Additionally, the PNIPAM-*b*-PANI drop shows a clear aggregation during irradiation, as can be seen in Figure S1I. 

The high solubility of the block copolymer in common solvents allows obtaining thin films PNIPAM-*b*-PANI by depositing drops of its solution, followed by solvent evaporation. By measurement of the electrical resistance of the films deposited on an interdigitated electrode and estimating the film thickness from the deposited mass, the resistivity value for the molecular nanocomposite is found to be of ca. 5.3 × 10^−4^ Ω^−1^ cm^−1^. The value is significantly lower than that of pure PANI (~1 Ω^−1^ cm^−1^) [38,39] but it is in the order of PANI statistical copolymers with substituted anilines (10^−3^–10^−4^ Ω^−1^ cm^−1^) [44,45] or blends with dielectric polymers [46]. The microstructure of blends should be similar to that of the copolymers’ films. Indeed, a blend of 5% PANI in PVA showed conductivities in the same order than the block copolymer [47]. Moreover, the conductivity could be increased by changing the deposition procedure. Since it was not the goal of the work, no attempt was made to optimize the conductivity. However, the conductivity values measured are only two orders of magnitude lower than carefully optimized CP loaded PNIPAM based membranes [48]. The result suggests that the PANI blocks are electrically connected in the solid state, but forming a composite while PNIPAM blocks act as an insulating matrix. 

**Figure 6:**
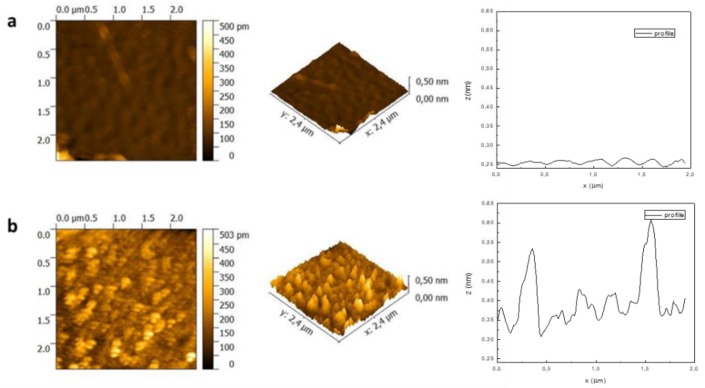
Atomic Force Microscopy (AFM) images of PNIPAM-*b*-PANI thin films: (**a**) before and (**b**) after NIR irradiation.

The morphological changes, upon NIR irradiation, of deposited thin films on a mica surface were studied by Atomic Force Microscopy (AFM) (Figure 6). A clear change of topography is observed after 2 min of NIR irradiation since the smooth surface of the deposited copolymer becomes populated by nanometrically sized globules. The NIR absorbing block (PANI) absorbs light and heat up by non-radiative decay. When the temperature overcomes the LCST of the thermosensitive (PNIPAM) block, inducing the coil to globule transition of PNIPAM forming arrays of globular shaped aggregates on the surface. The transition face of PNIPAM block can be observed in the film because it contains some retained water. A typical line profile (Figure 6, right) clearly shows the topographical changes upon irradiation. The small undulations (with a mean value of 0.03 nm) in the non-irradiated copolymer film (upper profile) become dominated by larger features (ca. 0.2 nm of size) upon NIR irradiation. 

This effect could be useful for the application of the material in photothermally responsive surface or smart coatings with control of the hydrophobicity properties by remote irradiation [49].

## 4. Conclusions

The results demonstrate that a copolymer containing a thermosensitive and a conjugated/conductive block induce a phase transition by the photothermal effect of light absorbed by the conjugated block. 

The thermoresponsive and conductive block copolymer can be obtained by a simple synthetic method involving two different successive polymerization reactions. The structure of the copolymer is ascertained by FTIR and NMR spectroscopy. The molecular weight and mean composition of the copolymer is evaluated using a combination of SLS and UV-visible spectroscopy.

PNIPAM-*b*-PANI turned out to be soluble in a wide range of organic solvents and water, unlike PANI. It was demonstrated that the block copolymer maintain the l properties of both blocks: the thermosensitivity of PNIPAM (with a LCST ~34 °C) and the electronic absorption/conductivity of PANI (directly dependent on the pH). Moreover, upon NIR irradiation, the PANI block absorbs the light and heat up, triggering the phase transition of the PNIPAM block. Such a transition provokes a morphologic change of the copolymer deposited as film and is detected by AFM. The controlled change at a distance using NIR light makes the copolymer a promising photothermal therapy agent since NIR light could penetrate several centimeters in biological tissue. 

Thin films obtained by deposition show electrical conductivity, revealing the presence of active PANI moieties. 

Besides the interesting properties of PNIPAM-*b*-PANI, the synthetic method could be applied to produce other block copolymers where either the conductive block (e.g., pH sensitive aminobenzoic acids) [50] or the vinylic block (e.g., pH sensitive polyacrylic acid) [51] have different properties. All the mentioned properties make the synthesized smart copolymer promising for diverse applications (e.g., photothermal devices, surfaces and coatings, and resistive sensors).

## Figures and Tables

**Table 1:** Solubility test for PNIPAM-*b*-PANI in different solvents at 20 °C.

Solvent	Solubility
1-octanol	Insoluble
Acetone	Soluble
Acetonitrile	Soluble
Carbon tetrachloride	Insoluble
Chloroform	Insoluble
Cyclohexane	Insoluble
Cyclohexanol	Slightly soluble
Dichloromethane	Insoluble
Dimethyl sulfoxide	Soluble
Ethanol	Soluble
Formic acid ^#^	Soluble
Isopropyl alcohol	Soluble
*N*-methyl-2-pyrrolidone ^#^	Soluble
Pyridine	Soluble
Tetrahydrofuran	Slightly Soluble
Water	Soluble

^#^ PANI is soluble in these solvents.

## Supplementary Materials

The following are available online at https://www.mdpi.com/2073-4360/11/11/1744/s1, Figure S1: Optical photographs of a copolymer solution drop under NIR at different irradiation times.
