# Supplementary material for: Synthesis of a Smart Conductive Block Copolymer Responsive to Heat and Near Infrared Light
Source: Polymers (Basel). 2019 Oct 24;11(11):1744. doi: 10.3390/polym11111744 (PMC6918231; doi:10.3390/polym11111744)
Supplement: Supplementary file 1 [file polymers-11-01744-s001.pdf]

Synthesis of a smart conductive block copolymer responsive to heat and Near Infrared light.

S. Bongiovanni Abel[1,†], K. Riberi[1], C. R. Rivarola[1], M. Molina[1]and C. A. Barbero[1]*

[1] Research Institute for Energy Technologies and Advanced Materials (IITEMA), National University of Río Cuarto (UNRC)-National Council of Scientific and Technical Research (CONICET). Ruta Nacional N° 36, Km 601, 5800, Río Cuarto (Córdoba), Argentina.

Electronic Supplementary information

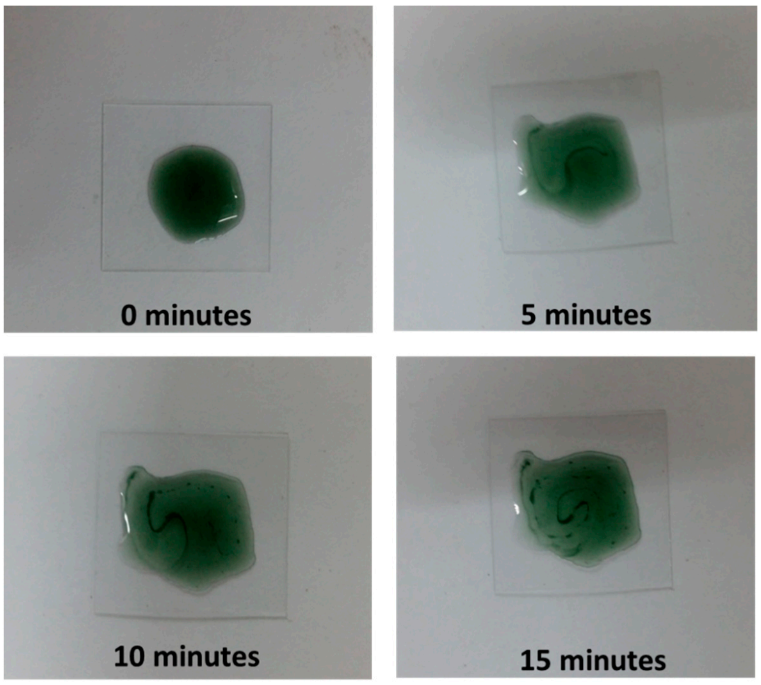

Figure S1. Optical photographies of a copolymer solution drop under NIR at different irradiation times.
